# Detection of Cannabinoids in Oral Fluid Specimens as the Preferred Biological Matrix for a Point-of-Care Biosensor Diagnostic Device

**DOI:** 10.3390/bios14030126

**Published:** 2024-02-27

**Authors:** Călin Trif, Dorin Harpaz, Evgeni Eltzov, Yardnapar Parcharoen, Chiravoot Pechyen, Robert S. Marks

**Affiliations:** 1 Avram and Stella Goldstein-Goren Department of Biotechnology Engineering, Faculty of Engineering Sciences, Ben-Gurion University of the Negev, Beer-Sheva 84105, Israel; trif@post.bgu.ac.il; 2 Institute of Biochemistry, Food Science and Nutrition, Faculty of Agriculture, Food and Environment, The Hebrew University of Jerusalem, Rehovot 76100, Israel; dorin.harpaz@mail.huji.ac.il; 3 Department of Postharvest Science of Fresh Fruit, Volcani Center, Agricultural Research Organization, Rishon LeZion 7505101, Israel; eltzov@volcani.agri.gov.il; 4 Chulabhorn International College of Medicine, Thammasat University, Klong Luang 12120, Pathum Thani, Thailand; yardnapa@tu.ac.th; 5 Center of Excellence in Modern Technology and Advanced Manufacturing for Medical Innovation, Thammasat University, Klong Luang 12120, Pathum Thani, Thailand; cpechyen@tu.ac.th; 6 Department of Materials and Textile Technology, Faculty of Science and Technology, Thammasat University, Klong Luang 12120, Pathum Thani, Thailand; 7 The Ilse Katz Center for Meso and Nanoscale Science and Technology, Ben-Gurion University of the Negev, Beer-Sheva 84105, Israel

**Keywords:** cannabis, cannabinoids, oral fluid, diagnostics, point-of-care, biosensors

## Abstract

An increasing number of countries have started to decriminalize or legalize the consumption of cannabis for recreational and medical purposes. The active ingredients in cannabis, termed cannabinoids, affect multiple functions in the human body, including coordination, motor skills, memory, response time to external stimuli, and even judgment. Cannabinoids are a unique class of terpeno-phenolic compounds, with 120 molecules discovered so far. There are certain situations when people under the influence of cannabis may be a risk to themselves or the public safety. Over the past two decades, there has been a growing research interest in detecting cannabinoids from various biological matrices. There is a need to develop a rapid, accurate, and reliable method of detecting cannabinoids in oral fluid as it can reveal the recent intake in comparison with urine specimens, which only show a history of consumption. Significant improvements are continuously made in the analytical formats of various technologies, mainly concerning improving their sensitivity, miniaturization, and making them more user-friendly. Additionally, sample collection and pretreatment have been extensively studied, and specific devices for collecting oral fluid specimens have been perfected to allow rapid and effective sample collection. This review presents the recent findings regarding the use of oral fluid specimens as the preferred biological matrix for cannabinoid detection in a point-of-care biosensor diagnostic device. A critical review is presented, discussing the findings from a collection of review and research articles, as well as publicly available data from companies that manufacture oral fluid screening devices. Firstly, the various conventional methods used to detect cannabinoids in biological matrices are presented. Secondly, the detection of cannabinoids using point-of-care biosensors is discussed, emphasizing oral fluid specimens. This review presents the current pressing technological challenges and highlights the gaps where new technological solutions can be implemented.

## 1. Introduction

### 1.1. Why Is It Important to Perform Cannabinoids Detection?

**Figure 1:**
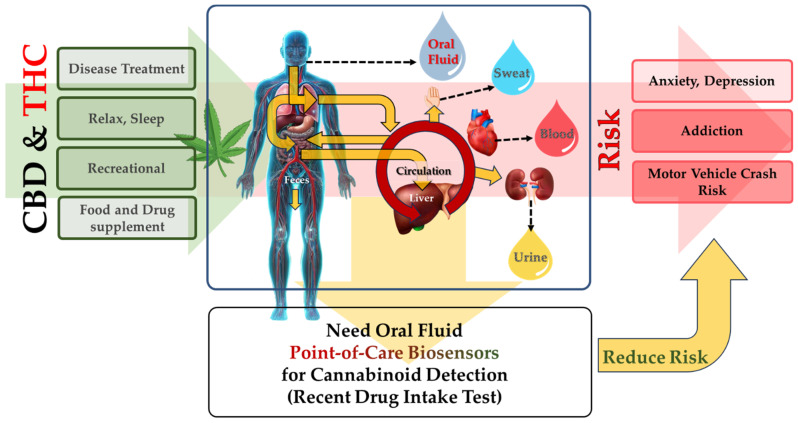
Cannabinoids route inside the human body after consumption.

An increasing number of countries have started to decriminalize or legalize the consumption of cannabis for recreational and medical purposes [1,2,3]. The active ingredients in cannabis affect multiple functions in the human body, including coordination, motor skills, memory, response time to external stimuli, and even judgment [4,5,6]. Therefore, there are certain situations when people under the influence of cannabis may be a risk to themselves or the public safety. Such situations include, for example, during driving, while operating heavy machinery, in specific workplace settings, in the army, in rehab programs for overcoming addictions, and during parental custody [7,8]. Over the past two decades, there has been a growing research interest in the detection of cannabinoids from various biological matrices with the need to develop a rapid, accurate, and reliable method of detecting cannabinoids in oral fluid (Figure 1), as it can reveal the recent intake in comparison with urine specimens, which only shows a history of consumption [9,10].

### 1.2. Different Types of Cannabinoids

Cannabinoids are a unique class of terpeno-phenolic compounds [11], with 120 molecules discovered so far [12]. They have been extensively studied after the initial discovery of Δ^9^-tetrahydrocannabinol (THC), which is the main compound responsible for the psychoactive effect of cannabis inebriation [13,14,15]. Based on the chemical formulas of cannabinoids, 11 subclasses have been distinguished. Besides THC, particular importance is also given to cannabidiol (CBD), which does not produce any psychedelic effects [16]. CBD is commonly used to treat various medical conditions in the human body [5,17,18,19,20,21,22,23,24,25,26,27,28,29,30]. In addition to THC and CBD, several other cannabinoids have already been identified as having medicinal properties (e.g., tetrahydrocannabivarin (THCV), cannabichromene (CBC), cannabigerol (CBG), and cannabinol (CBN)) [31]. Other types of cannabinoids include the endocannabinoids that are produced in the human body (e.g., anandamide, 2AG) [32], which regulate a vast number of physiological functions, such as the immune responses, cognition, emotions, motor coordination, body temperature, and sleep/wake cycle [32,33,34].

### 1.3. Preferred Cannabinoids as Targets for Detection

THC is still the most well-studied cannabinoid. In 1974, Edward R. Garrett et al. initially investigated the physico-chemical properties, bonding capabilities, adherence to different materials, and solubility of THC [35]. The reported solubility of THC in water is 2.8 mg/L, while in a saline solution (0.15 M NaCl), it is lower, at 0.77 mg/L, at a temperature of 23 °C. Different organic solvents affect the solubility of THC while also providing good stability, especially in the extraction process. Other findings report the adherence of THC to glass, in a proportion of 20–40%, based on its concentration in an aqueous-ethanol solution (0.1–0.05 µg/mL) and the contact surface. In contrast, the adherence of THC to plastic and rubber caps that were used to seal the plastic tubes ranged between 70–97%. In order to remove or reduce the adherence of THC to these materials, the sample can be first pretreated with silyl. Most importantly, the study showed that THC had a high rate of protein binding of up to 97%, which was not dependent on its concentration [35]. It was previously reported that once THC reaches the bloodstream, it binds proteins, and as a result, it is transported to various tissues and organs [36]. Once the THC reaches the blood, only a small portion remains free after it is rapidly absorbed into adipose tissues, and then it is released gradually over time [37]. It is known that THC is rapidly degraded in an acidic solution; therefore, if it is administered orally, it is degraded in the stomach, and only a small portion is bio-available, as compared to smoked or vaporized administration [38].

### 1.4. Existing Challenges in the Detection of Cannabinoids

Significant improvements are continuously made in the analytical formats of various technologies, mostly concerning improving their sensitivity and miniaturization and making them more user-friendly [39]. Additionally, sample collection and pretreatment have been extensively studied, and specific devices for collecting oral fluid specimens have been perfected to allow rapid and effective sample collection [40]. The main challenge that is increasingly discussed is the influence of the interactions between different cannabinoids and with different substrates and materials, primarily THC as the most studied cannabinoid. For example, it has been previously shown that THC interacts with plastic containers [35], making the extraction of cannabinoids from collection devices a challenging process, especially without the use of buffers. This implies that the use of certain analytical procedures is currently limited and that more research should be conducted in order to evaluate any possible errors due to the potential loss of THC. Moreover, efforts are being made to improve the interpretation of the results to allow a more accurate evaluation [41]. The interpretation of results is a crucial step for detecting the actual levels of cannabinoids in the human body, which further leads to better decision-making processes by the authorities. The pressing current challenge is to develop a diagnostic technology that demonstrates high sensitivity and specificity and, ideally, does not need chemicals in the sample preparation process. This review presents the recent findings regarding the use of oral fluid specimens as the preferred biological matrix for cannabinoid detection in a point-of-care biosensor diagnostic device. A critical review is presented, discussing the findings from a collection of review and research articles, as well as publicly available data from companies that manufacture oral fluid screening devices. Firstly, the various conventional methods used to detect cannabinoids in biological matrices are presented. Secondly, the detection of cannabinoids using point-of-care biosensors is discussed, emphasizing oral fluid specimens. This review presents the current pressing technological challenges and highlights the gaps where new technological solutions can be implemented.

## 2. Detection of Cannabinoids

**Figure 2:**
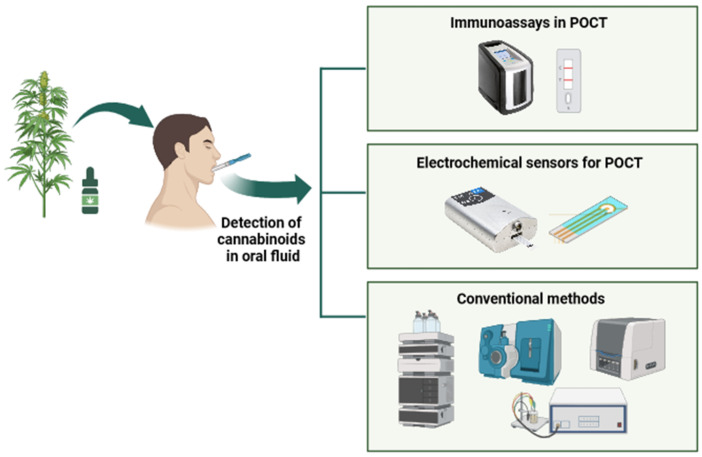
Detection of cannabis from oral fluid specimens.

Figure 2 shows the detection of cannabis from oral fluid specimens. These techniques require multiple steps in traditional protocols, including specimen collection, stabilization, extraction, screening analysis, confirmation, and interpretation of results. Efforts are taken to simplify the procedure, such as simultaneous stabilization and extraction. Chromatographic procedures such as GC-MS, LC-MS, and HPLC-MS are considered the gold standard due to their precision and quantitative capabilities. Immunoassays, notably ELISA and EIA, are very sensitive but produce qualitative results that require extra validation steps. Electrochemical sensors are gaining popularity for point-of-care testing due to their portability and quick findings. However, they are still under development and have issues with selectivity and integration into user-friendly equipment.

### 2.1. Conventional Detection Procedure

The analytical detection method must be validated, well-known, and accepted as a standard diagnostic device. In the case of detection of cannabinoids in biological matrices, for example, oral fluid specimens, certain steps must be followed: (1) collection of the biological specimen; (2) stabilization of the cannabinoids in buffers; (3) extraction of the cannabinoids from the collection device; (4) screening analysis of the extract; (5) confirmation analysis with quantification of the cannabinoids; and (6) the interpretation of the results. Despite the importance of each step, in order to expedite the process of detecting cannabinoids, fewer steps should be defined. For example, the stabilization and extraction of cannabinoids in the buffer can be conducted in parallel in the collection device. An additional contribution to the long detection process of cannabinoids is the need to send a second sample for validation to a centered lab after a positive result is detected. However, this process is time-consuming and requires collecting a second sample that can differ from the first one, especially when quantitation is required. Several conventional techniques are currently used for the detection of cannabinoids (Table 1), which can be categorized into either chromatographic methods or immunoassays. Additional methods include electrochemistry and capillary electrophoresis, but these techniques are still mostly research-based [42,43]. The gold standard lab-based methods for cannabinoid detection are based on chromatography. These include gas chromatography coupled with mass spectrometry (GC-MS), liquid chromatography coupled with mass spectrometry (LC-MS), and high-performance liquid chromatography coupled with mass spectrometry (HPLC-MS), which can also be coupled with MS for improved detection (HPLC-MS-MS) [44]. These techniques are extremely specific, sensitive, and quantitative. Not only that, but these techniques can analyze various biological matrices for cannabinoid detection with great accuracy. They must also be used in order to validate the results that are obtained by an on-site testing device.

### 2.2. Immunoassays in Point-of-Care Biosensors

The most common method for the detection of cannabinoids in oral fluid specimens is immunoassay screening [45]. This offers a promising approach for point-of-care detection of cannabinoids in oral fluid due to its high specificity, sensitivity, and potential for rapid results. These assays rely on the specific binding of antibodies to target molecules, such as THC and its metabolites [46]. In the context of cannabinoid detection, an antibody specific to the target cannabinoid (THC, THC-COOH, and CBD) is immobilized on a solid support [47]. The oral fluid sample is then introduced, allowing cannabinoids to bind to the immobilized antibody. Unbound material is washed away, and a secondary antibody labeled with a detectable signal (e.g., enzyme, fluorescent molecule) is introduced [48]. This secondary antibody binds to the first antibody if the target cannabinoid is present, creating a signal proportional to the quantity of bound cannabinoids. The intensity of this signal, measured visually or electronically, allows for qualitative or quantitative detection of cannabinoids in oral fluid. These techniques are widespread and widely used in a variety of domains. In the lab, enzyme-linked immunosorbent assay (ELISA) [45] or enzyme immunoassay (EIA) [49] are used in order to detect cannabinoids with good sensitivity and specificity. They have the advantage of being easily modified for lateral flow immunoassay, thus allowing them to be used on-site as point-of-care devices [40]. The benefits of point-of-care biosensors include accessibility, portability, robust setup, rapid detection procedure, and ease of use by non-expert personnel. Immunoassays are based on the formation of immunocomplexes that are conjugated to signaling particles after the analyte of interest binds to the specific antibodies in the test, and a measurable signal is produced (e.g., colorimetric or fluorescent) [50]. Various approaches are used in order to improve the sensitivity of the immunoassays, for example, by color enhancement, amplification of the immunocomplex response, or by developing new signaling nanoparticles [48]. Several different types of readers have been developed for the improved interpretation of the detection lines. They are based on projecting light at certain wavelengths on the test strip and then collecting the reflectance of the reporting particles by photodiodes, which convert the photon emission into a digital signal [51]. Another more popular technique is the use of simple cameras, even from a smartphone, to interpret the color intensity of the strip lines [52]. The existing immunoassays are effective but still have disadvantages [45]. Most importantly, these methods are not fully quantitative, with only a few achieving semi-quantitation [53], for example, after identifying a THC cut-off of 4 ng/mL [54], allowing detection in the range of 4–200 ng/mL [55]. Another significant disadvantage is the need for an additional sample collection device, where the sample is usually diluted in special buffers in order to improve stability and eliminate unspecific binding of THC to plastic or glass surfaces [56,57]. This method does not allow the reuse of the same sample for multiple testing; thus, two samples need to be collected, one for the on-site screening and another to be later sent to the laboratory for validation. In addition, another important aspect that must be considered is the need for an analytical device capable of detecting small molecules as the test target analyte, which is a challenge in developing new technologies.

### 2.3. Electrochemical Sensors for Point-of-Care Testing

While immunoassays remain the most used POCT technique for the detection of cannabinoids in oral fluid, there is a growing interest in exploring electrochemical sensors due to their effectiveness, portability, and rapid results [58]. The specific details may vary depending on the type of sensor, yet the general principle of how most electrochemical sensors work for cannabinoid detection in oral fluid involves several steps: (1) capturing the analyte on a sensor chip which has a modified surface with a recognition element, such as an aptamer or antibody, specifically designed to bind to the target cannabinoid molecule present in the oral fluid sample; (2) interaction, where cannabinoids come into contact with the sensor chip, and the target molecule binds to the recognition element on its surface; (3) signal generation, when the binding event alters the electrical properties of the sensor surface in different ways, determining a change in the conductivity, resistance, or difference of electrical potential; (4) measurement and analysis, where the sensor measures the resulting change in electrical properties (conductivity, resistance, or current) and converts it into a digital signal, which is then analyzed by an electronic device to determine the presence and concentration of the target cannabinoids; and (5) output, where the analysis software interprets the signal and provides information about the presence and concentration of the cannabinoids. Several types of electrochemical sensors have been researched for the detection of cannabinoids in oral fluid, with the use of aptamers or nanoparticles, conductometric, and impedimetric sensors [59]. They all hold promise for POC cannabinoid detection, but they are still under development and face challenges like selectivity, sensitivity, and integration into user-friendly devices. Aptamers and nanoparticle-based sensors show potential but require further research and optimization for oral fluid applications [60,61]. Conductometric and impedimetric sensors are simpler but face limitations in specificity and require miniaturization for POC suitability [62,63]. To date, there are no commercially available electrochemical sensors specifically designed for cannabinoid detection in oral fluid. While research and development in this area are active, these sensors have not yet received regulatory approval and widespread market adoption for POC testing.

## 3. Biological Matrices for the Detection of Cannabinoids

### 3.1. Various Biological Matrices, including Blood and Urine Specimens

**Figure 3:**
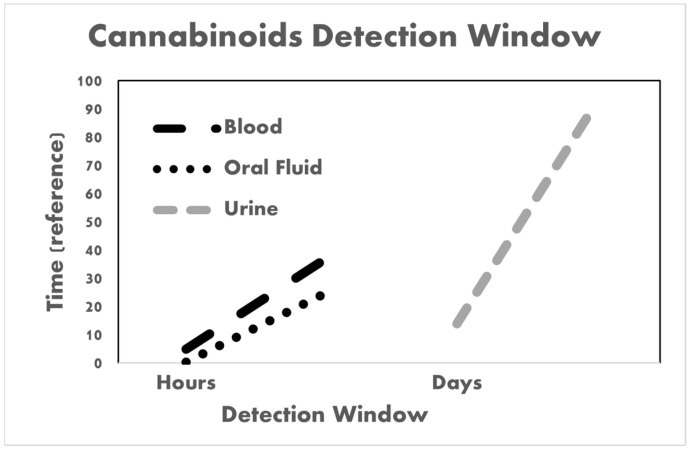
Detection window of cannabinoids in urine, blood, and oral fluid specimens.

Various biological matrices are used for the detection of cannabinoids, with the choice between them being dependent on the required application (Table 2). Several factors are taken into consideration before choosing the biological matrix, including the applied analytical method [41,44,64], its sensitivity, the amount of sample required for analysis, the time range for detection [65], the time since the latest cannabinoid consumption, the availability of the matrix, and the need for analysis at point-of-care or in a central lab. In the latter case, a collection device is also required, which includes buffers for stabilization [40,56,66]. Different biological matrices have different characteristics. Blood is considered the reference standard biological matrix because it shows the analyte levels in the internal general circulation and allows the derivation of correlations between its quantity and the levels of impairment as well as the time period since the latest usage [67,68]. Moreover, the analytical methods used to detect cannabinoids in the blood are validated without discrepancies between laboratories [39]. However, the blood sample collection requires trained personnel and is invasive; therefore, it can be unpleasant for the subject. It is also possible that the donor will refuse to provide his blood sample. Urine is one of the biological matrices that has been heavily researched [65,69,70,71,72,73,74,75,76,77,78,79,80,81,82,83,84,85,86,87,88,89,90,91,92]. The analytical methods for the detection of cannabinoids in urine samples have also been validated and have good sensitivity and specificity; however, the screening of cannabinoids in urine samples is limited to THC-COOH, which is a metabolite that is created after the enzymatic degradation of THC [93,94]. Another disadvantage of the use of urine samples is that it is not a good indicator of recent cannabis intake but rather only of the history of usage. A urine sample is considered to be relatively easy to collect from the subject, even though it requires a special room, and for the collector personnel to be of the same sex in order to protect the privacy of the subject and also to eliminate the existing risk of matrix adulteration. Other biological matrices include hair, sweat, and even nails; however, only a few analytical methods have been developed for the detection of cannabinoids in these types of specimens [37]. These biological matrices are mainly useful in detecting the history of cannabinoid use because they do not show the recent intake or the last time of consumption, meaning they are not suitable for point-of-care [50]. Moreover, they are often not used for the detection of cannabinoids because they are exposed and, therefore, can be easily contaminated by the environment, which requires a long and difficult sample preparation [95]. In addition, the detection window varies depending on the analytical method and the biological matrix (Table 3, Figure 3). In these studies, both inter and intra-subject differences were observed. The detection window depends on several factors, including the analyte, time of consumption, dosage, metabolism, excretion of the drug, and other physiological factors.

### 3.2. Oral Fluid as the Preferred Biological Matrix

In recent years, increasing research efforts have focused on using oral fluid specimens as the matrix of choice for cannabinoid detection [36,41,44,73,86,103,104,105,106]. It is relatively easy and fast to collect an oral fluid specimen, and it can be done on-site under direct supervision, eliminating any chances of an adulterated sample. Various analytical methods were developed for the detection of cannabinoids in oral fluid specimens, which are similar to methods for detection in urine; however, because of the differences in the matrix composition, several changes were made over the years in order to improve their accuracy. Moreover, oral fluid specimens are the preferred choice in order to detect recent cannabis intake. In this case, the analyte of interest is the parent drug, THC. Due to the direct contamination of the oral cavity with THC during smoking, significantly high values of the analyte can be found in the first hour after consumption [107]. In addition, it is important to note that cannabinoids bind to proteins to a high degree [35]. For example, unbound THC molecules are absorbed directly into adipose tissues or organs from the blood. Then, through diffusion, THC–protein complexes can pass from the blood into the oral fluid. The pH differences between the blood and the oral fluid keep the THC–protein complexes in the salivary glands. In order for the THC–protein complexes to reach the oral fluid, they need to transfer through five identified barriers, including the capillary wall of the blood vessels, the interstitial space between the capillary wall and the cell membrane of acinar cells, the membrane of the acinar cell, the fluid inside the acinar cell, and the luminal cell membrane [108]. Previous studies investigated the correlations between the cannabinoid concentrations in oral fluid and those in the blood/plasma. Xu C. et al. [44] examined 4080 subjects between 2007 and 2010, and the samples were analyzed using UPLC-MS and HPLC-MS. A correlation was reported between the two matrices in a proportion of up to 90%. Then, in 2014, Gjerde H. et al. [109] compared the two matrices and reported a correlation between them. However, high intra- and inter-subject variability was reported, especially in the first hour after the smoking of cannabis because of the direct contamination of the buccal cavity. The main parameter that influences the correlation between the two matrices is the oral administration of cannabis, which is not yet common worldwide, except for a few countries that have legalized food products that contain cannabis, such as brownies or space cakes [110]. It is not yet fully understood whether the cannabinoid concentrations in the blood can be predicted from the analysis of oral fluid specimens.

### 3.3. Collection of Oral Fluid Specimens for Cannabinoids Recovery

Properly collecting the oral fluid sample is the first step toward a successful detection analysis. This step involves the collection of the saliva in a special container, usually made from plastic. There are three main methods by which an oral fluid specimen can be collected: passive drool, expectoration, and collection via a collection device (Table 4). The most common method with the best results is the collection via a collection device. This method is officially approved and offers improved sample stability due to the addition of buffers. In general, buffers contain antioxidants to counteract free radicals that can degrade the cannabinoids and pH stabilization compounds such as phosphate [111]. Another aspect to consider is that buffers reduce oral fluid viscosity, improving measurement accuracy and diluting analyte concentrations [66]. Most of the collection devices are made up of a container, pad, buffer, and volume adequacy indicator that changes its color once the specified volume of oral fluid is collected. The sample collection is fast, usually taking around 1–2 min. The pad also acts as a filter for the oral fluid, allowing only liquid absorption. Newer devices demonstrate more homogeneity (Table 5). An important aspect to be considered is the amount of analyte that is recovered from the collection device. When diluting the analyte with buffer, the sample’s stability is increased, and there is a lower loss of analyte. However, the dilution factor has to be taken into consideration, and the analytical method of choice should allow increased sensitivity. In addition, THC is easily absorbed into plastic and requires the use of a solvent to remove it completely. However, some collection devices do not use a buffer and realize poor analyte recovery.

### 3.4. Stability of Cannabinoids within Oral Fluid Specimens

Generally, the integrity of oral fluid specimens is better than that of urine samples [92]. However, oral fluid specimens are still influenced by multiple parameters. The effects of tobacco, different foods, orange juice, coffee, soymilk, chewing gum, mouthwash, toothpaste, vinegar, and two commercial products specifically made for adulterating the oral fluid matrix were investigated for potential interference [129]. Only vinegar produced one false-positive result from all the tested parameters [41]. In addition, it was previously reported that bicarbonates excreted from salivary glands into the oral fluid offer good buffer properties for containing the cannabinoids [104]. The only problem could be this collection methodology does not use surfactants, thus allowing the cannabinoids to bind to the plastic container, which may then result in the poor recovery of the analyte [115]. The stability of cannabinoids within a biological matrix also depends on several factors, such as buffers, containers, temperature, and exposure to light [35]. Lee D. et al. [130] investigated the stability of cannabinoids within an authentic oral fluid. It was reported that the expectorated oral fluid degraded quickly at room temperature and that the percentage of THC was reduced by half after 10 days. The maximum storage time was found to be 24 weeks for THC fortified in the Quantisal buffer at −20 °C. However, when buffers were used, the recovery of cannabinoids after 28 days ranged from 78 to 118%, depending on the oral fluid collection device [36]. The ability to reuse the same sample for multiple analyses would allow the detection in multiple places or with multiple techniques. Even though the samples can be collected one after another, there were cases when the results were different, or the sample volume was insufficient. The detection process starts with collecting one sample that is then analyzed. Based on the results, when a positive result is obtained, another sample is only then collected and sent to the lab for confirmation. However, the process can become complex and time-consuming when several samples are required. For this purpose, one collection device was developed for collecting two clean oral fluid samples simultaneously, in a two-split tube [131]. Except for the cases of spectrophotometric detections, none of the currently used devices can reuse the sample for multiple analyses. The analytical methods are different and require a specific sample volume for the detection analysis.

## 4. Detection of Cannabinoids in Oral Fluid Specimens in Lab-Based Techniques and on-Site Point-of-Care Biosensors

The detection of cannabinoids in lab-based techniques is more accurate and sensitive but significantly slower than that in on-site point-of-care biosensors. In addition, on-site point-of-care biosensors usually allow the detection of a single cannabinoid, while the lab-based techniques provide detection results for multiple cannabinoids and their metabolites in parallel. While it may seem preferable to send a sample for cannabinoid detection to a centered lab, in certain circumstances, there is a need for an on-site result. In order to correctly calculate the concentration of the cannabinoids in oral fluid specimens, an accurate volume of approximately 1 mL of an oral fluid specimen is usually collected. Then, after taking into account the additional known buffer volume, the exact concentration can be calibrated for each on-site point-of-care biosensor system [132]. For example, in the case of the StatSure device, the following formula is used:

$$C_{corrected}=\frac{C_{uncorrected}*(1+w-\underline{w})\;}{2*(w-\underline{w})}$$

The variables within the formula include $\underline{w}$ = average weight of empty StatSure device; *w* = weight of sample and StatSure device; *C_uncorrected_* = uncorrected concentration of analyte; *C_corrected_* = concentration of analyte corrected for volume of oral fluid collected. Then, the density of the mixture of the oral fluid specimen and the buffer is assumed to be 1 g/mL. Additional measurable parameters are determined, including sensitivity, specificity, accuracy, prevalence, and predictive values (positive or negative). Most biosensor systems for cannabinoid detection show a positive result only above a certain predetermined cutoff value. Another important concern in cannabinoid detection is cross-reactivity. For example, anti-THC antibodies also react with different concentrations of THC-COOH, CBD, and CBN [49]. The detection of cannabinoids in both lab-based techniques (Table 6) and on-site point-of-care biosensors (Table 7) is presented, with the description of the technique as well as the levels of the cannabinoids.

## 5. Conclusions

This review presents the recent findings regarding the use of oral fluid specimens as the preferred biological matrix for cannabinoid detection in a point-of-care biosensor diagnostic device. Currently, cannabinoids are detected in various biological matrices, including blood, oral fluid, urine, sweat, breath, and hair. The detection window varies depending on the analytical method and the biological matrix used. It is also dependent on several factors, including the analyte, time of consumption, dosage, metabolism, excretion of the drug, and other physiological factors. The detection of cannabinoids in oral fluid and blood specimens provides information on the most recent drug intake, with a detection window between 15 min and up to 24 h in single-use cases. The other most well-studied and used biological matrix, urine, provides information only on the history of use, with a detection window of 14–90 days. Oral fluid is the preferred biological matrix to be used for detection in point-of-care biosensors since blood is a much more complex specimen that requires pretreatment and specialized analytical devices. Moreover, individuals are still entitled to refuse to provide blood samples, as compared to oral fluid specimens, which makes the screening of drug use more difficult to implement. In addition, various conventional techniques and point-of-care biosensors were also reviewed in this study. The details discussed highlight their detection sensitivity and ability for on-site drug use screening in oral fluid specimens. Detection of synthetic cannabinoids in oral fluid is still challenging due to the high number of new compounds that do not work with current technology. Additionally, these synthetic cannabinoids are sometimes mixed with cannabimimetic compounds, which are an entirely new class of substances that need to be detected. Regardless of the challenges, efforts are being made to detect and control such substances by creating POCT devices utilizing cross-reactivity for a class of compounds or better collection devices for the ability to take samples to a full-scale laboratory. With improvements to the current technologies for on-site detection of psychoactive compounds, the accuracy can be improved, and a correlation between the physiological concentration of cannabinoids and levels of impairment is still needed. A joint venture between Biosensorix in Singapore and Eclipsedx in the USA are developing cannabinox, a device able to detect cannabis bioactive compounds, including in saliva [164]. To conclude, this review presents the current pressing technological challenges and highlights the gaps where new technological solutions can be implemented.

## Figures and Tables

**Table 1:** Conventional analytical techniques for the detection of cannabinoids in biological matrices.

Analytical Technique	Sensitivity	Blood	Oral Fluid	Urine	Sweat	Breath	Hair	Point-of-Care
Immunoassay	High	**Yes**	**Yes**	**Yes**	**Yes**	No	**Yes**	Yes
General spectrophotometry (ultraviolet, infrared, fluorescence, visible)	Low	No	**Yes**	No	No	No	No	Yes
Raman	Moderate	No	**Yes**	No	**Yes**	No	No	Yes
Nuclear magnetic resonance	Moderate	**Yes**	No	**Yes**	No	No	No	No
Gas chromatography-mass spectrometry	High	**Yes**	**Yes**	**Yes**	**Yes**	**Yes**	**Yes**	Not yet
Liquid chromatography-mass spectrometry	High	**Yes**	**Yes**	**Yes**	**Yes**	No	**Yes**	Not yet
High-performance liquid chromatography-mass spectrometry	High	**Yes**	**Yes**	**Yes**	**Yes**	**Yes**	**Yes**	No
Isotope ratio mass spectrometry	Low	No	No	**Yes**	No	No	No	No
Thin-layer chromatography	Low	**Yes**	**Yes**	**Yes**	No	No	No	No
Gas chromatography–nitrogen phosphorous detector	Moderate	**Yes**	**Yes**	**Yes**	**Yes**	No	No	No
Gas chromatography–flame ionization detector	Low	**Yes**	**Yes**	**Yes**	**Yes**	**Yes**	No	No
Liquid chromatography–ultraviolet detector	Low	**Yes**	No	**Yes**	No	No	No	No
Chemiluminescence	High	**Yes**	**Yes**	**Yes**	**Yes**	**Yes**	**Yes**	Not yet
Electrochemical detector	High	**Yes**	**Yes**	**Yes**	**Yes**	**Yes**	**Yes**	Yes
Capillary electrophoresis	High	**Yes**	**Yes**	**Yes**	**Yes**	No	**Yes**	Yes
Supercritical fluid chromatography	Moderate	**Yes**	**Yes**	**Yes**	**Yes**	No	No	No

Detection ability of analytical devices for cannabinoids in various biological matrices, including blood, oral fluid, urine, sweat, breath, and hair. The sensitivity of the devices and whether they are suitable for use on-site as point-of-care devices are also specified. The bold and color highlight the techniques and specific matrix that can be used for POCT devices.

**Table 2:** Biological matrices for the detection of cannabinoids [37,41].

Biological Matrix	Advantages	Disadvantage	Cannabinoids Detection
Blood	Indicates recent intake Samples adulteration is eliminated Interpretation of results is easy and accurate	Sample collection is difficult and can be done only by trained personnel Short detection times, also dependent on the analyte concentration Analysis is complex, costly, and requires specialized laboratory Current technologies are not developed for point-of-care testing	Show recent drug intake
Urine	Relatively easy to collect High concentration of analytes Large sample volumes Provides a history of drug use between 2 days to several weeks Easy and inexpensive to analyze Suitable for workplace and compliance testing No need for sample pretreatment Point-of-care test kits are available	Sample is easy to adulterate Interpretation of results can be difficult Mostly contains metabolites and not parent drug; not suitable for detecting a recent intake Observed sample collection is considered a personal privacy violation	It does not show recent drug intake but offers a view of drug usage over the last month
Oral fluid	Easy to collect without the intrusion of privacy Can collect several samples Contains parent drug Difficult to adulterate No need for specialized personnel Suitable for the workplace, compliance, and forensic testing Point-of-care-test kits available Indicates recent intake New analytical techniques can be developed	High inter- and intra-subject variability Sample size limited Drug concentration may be low and subsequently difficult to analyze Interpretation of results is complex Requires confirmatory analysis with a very sensitive analytical method Cannabis derivatives do not pass easily from blood into saliva; therefore, potential sensitivity issues for general screening High values are registered in the 1st hour after consumption in the case of inhalation	If there is no direct contamination of the buccal cavity, it depends on the transfer of analytes from the blood into the oral fluid
Sweat	Easy to collect and non-invasive Point-of-care tests available Sweat collection patches for drug-use monitoring are available	Currently available test kits are expensive and not commonly used Small sample volumes Requires a very sensitive analytical method High chances of external contamination Interpretation of results is difficult	Sample can be easily contaminated from the environment
Hair	Provides a history of drug use Sample stable for years Sample collection is non-invasive Extremely useful for crime-related investigation and drug consumption	Can be easily contaminated from the environment Analysis requires specialized laboratory Not suitable for point-of-care testing Analysis is expensive Sample collection is dependent on the length of hair Interpretation of result is limited Can be influenced by dyeing and perming treatment	Does not show recent intake

**Table 3:** Detection window of cannabinoids in urine, blood, and oral fluid specimens.

Matrix	Analyte	Cutoff (ng/mL)	Use	Detection Times	Reference
Urine	THC-COOH	15	Single-use	<3 days	[37,65,96]
	THC-COOH		Moderate use—four times a week	<4 days	
	THC-COOH		Chronic use	14–90 days	
Blood	THC	10	Single-use	<5 h	[65,96]
	THC THC-COOH		Chronic use	<14 days	[96]
	THC-COOH		Single-use	<36 h	[65]
Oral fluid	THC CBD CBN THC-COOH	0.5	Single and chronic use	THC: 12–34 h CBD: 1–22 h CBN: 1–13.5 h	[96,97,98,99,100,101,102]
	CBG	1	Single-use	15 min	[101]
	THCV	0.4	Single-use	15 min	
	THCA-A	1	Single-use	<90 min	

Abbreviations: Δ^9^-tetrahydrocannabinol (THC); cannabidiol (CBD); cannabinol (CBN); cannabigerol (CBG); tetrahydrocannabivarin (THCV); tetrahydrocannabinolic acid (THCA).

**Table 4:** Collection methods of oral fluid specimens [41].

Method of Collection	Advantage	Disadvantage
Passive drool	Accurately reflects drug concentration Does not require special training and tools	Can be unpleasant for the donor Slow process Low drug stability without the use of buffers THC binds to plastic containers
Expectoration	Provides clean oral fluid, which can increase assay sensitivity Cheaper in comparison with other methods	Can be unpleasant for donors and collectors Contains impurities Samples need to be centrifugated before analysis Low recovery when collected in plastic containers and without the use of buffers Lower drug stability without the use of buffers Sample filtration is required when it is used in laboratory analysis
Salivary stimulation	Increased sample volume In case of dry mouth, is effective in collecting enough volume	Lower pH due to stimulation Increased bicarbonate and ions concentration Lower drug concentration
Collection device	Hygienic Requires little time for collection Standard collection devices have buffers inside the collection tube Sample collected in sponge or pad also filtrates the sample Newer collection devices include a volume adequacy indicator Multiple sample collections with the same properties	Buffers and surfactants can interfere with laboratory methods of screening Sample is diluted due to the use of buffers Can produce ion suppression or enhancement Recovery of cannabinoids may differ with the use of different buffers

**Table 5:** Description of collection devices of oral fluid specimens.

Device (Manufacturer, City and Country)	Components	Collection Method	Volume Indicator	Oral Fluid Volume (mL)	Extraction Technique	Tetrahydrocannabinol (THC) Recovery (%)	Refs.
Certus (Concateno, Corston, UK)	Pad, container, buffer (3 mL), volume adequacy indicator	Absorbent pad is inserted into the buffer	Yes	1	Pad placed in the buffer for 24 h at 4 °C	54 37–44 (71–85)	[112,113]
Cozart (Cozart Bioscience, Abingdon, UK)	Pad, container, buffer (2 mL), volume adequacy indicator	Absorbent pad is inserted into the buffer	Yes	1	Elute with a proprietary buffer	96 75.9 (6.2) 94.5 (0.02) 67.4	[114,115,116,117,118]
DCD 5000 (Dräger, Lübeck, Germany)	Cassette: tap, pad, container, buffer, volume adequacy indicator Device: reader, printer (electronic and printed results)	Absorbent pad is part of a device; buffer is added after collection	Yes	0.38	Placed in isopropanol for 1 h and centrifuged	89.8–93.8	[119,120]
DrugWipe 5, 5S, 6, 6S (Securetec, Neubiberg, Germany)	Cassette: collection pad, buffer, LFI strip	Sweep the tongue, saliva was collected by a change of color, not quantitative	No	-	-	-	[121]
Greiner (Greiner Bio-One GmbH, Greinerstraße, Austria)	Rinsing solution (6 mL), OF extraction solution (4 mL), collection beaker, 2 OF vacuum transfer tubes	Collection by thoroughly rinsing out the oral cavity (2 min), expectoration into collection beaker, transfer to Saliva Transfer Tubes, add stabilizers	No	Determined spectrophotometrically w/dye in the extraction solution	Determined spectrophotometrically w/dye in the extraction solution	73.6 (4.3)	[36,115]
Intercept (OraSure Technologies, Bethlehem, PA, USA)	Cotton fiber pad, plastic container, buffer (0.8 mL)	Absorbent pad is inserted into the buffer	No	1 mL max	Centrifuged to recover the buffer-oral fluid mixture Centrifuged to recover the buffer-oral fluid mixture Centrifuge, add 2 mL methanol to stabilization buffer and pad, incubate and shake 15 min, centrifuge	37.6 31.2–57.2 Additional 19.2–34.4 37.6 39.2	[115,116,122,123,124]
Quantisal (Immunalysis, Pomona, CA, USA)	Cellulose pad, plastic container, buffer (3 mL), volume adequacy indicator	Absorbent pad is inserted into the buffer	Yes	1 ± 0.1 (10%)	Buffer-oral fluid mixture separated with serum separator tube Pad placed in the buffer for 24 h at 4 °C	81.3–91.4 94 55.8 55.8 (12.0) 81.3–94.4 (4.8–12.1) 74–80 (12–16)	[44,57,112,113,115,125]
OraCol and OraCol Plus (Malvern Medical Developments, Worcester, UK)	Foam swab, microtube, centrifuge tube	Saliva is collected by rubbing the sponge swab firmly along the gum until the sponge is wet	No	1	Centrifugation with a tube inserted	<12.5	[36,115]
OraTect III (Branan Medical Corporation, Irvine, CA, USA)	Cassette: collection pad, LFI strip	Directly applied to the mouth	No	-	-	-	[126]
OraTube (Varian, Palo Alto, CA, USA)	Pad, plastic container, expresser	Absorbent pad	No	1.979 mL (in vitro)	-	-	[115]
Salicule (Acro Biotech, Montclair, NJ, USA)	Expectoration straw, container marker w/scale	Expectoration	Yes	-	-	-	[36]
Saliva-Sampler (StatSure Diagnostic System, Sterling, VA, USA)	Cellulose pad, plastic container, buffer (1 mL), volume adequacy indicator	Absorbent pad, buffer	Yes	1	Buffer-oral fluid mixture extracted from the pad with filter	85.4 65.5–68.1 85.4 (7.0) 100–106 (5–6)	[36,113,115,127]
Salivette (Sarstedt AG & Co., Nümbrecht, Germany)	Cotton swab, plastic container	Cotton swab is chewed, placed back into the container then centrifuged	No	Unknown	Centrifugation with a tube inserted	<12.5	[115,128]

**Table 6:** Detection of cannabinoids in oral fluid specimens in lab-based techniques.

Analytical Method	Collection Device	Oral Fluid (OF) Sample Volume	Extraction Method	Derivatization	Analytes Detected (µg/L)	Detection Range (µg/L)	Refs.
LC-MS	Plastic tube	200 µL of expectorated OF	Liquid-liquid extraction	None	THC: 2	THC: 2–250	[133]
LC-MS	Salivette	500 µL of Salivette OF	SPE	None	THC: 2	THC: 2–100	[134]
LC-MS/MS	Intercept	100 µL OF or 500 µL of Intercept OF	Liquid-liquid extraction	None	THC: 0.5 for 100 µL sample	THC: 0.5–100	[135]
GS-MS/MS	Intercept	100 µL of Intercept OF	SPE	HFIP and PFAA	THC-COOH: 10	THC-COOH: 10–240	[136]
2D-GC-MS	Quantisal	1 mL of Quantisal OF	SPE	HFIP and TFAA	THC-COOH: 2	THC-COOH: 2–160	[64]
GS-MS	Quantisal	Unspecified vol. of quantisal OF	SPE	BSTFA	THC: 0.5 CBD: 0.5 CBN: 1 THCAA	THC: 1–16 CBD: 1–16 CBN: 1–16	[137]
LC-QTOF-MS	Plastic tube	500 µL of synthetic OF	Liquid-liquid extraction	None	THC: 0.05, 0.1 THC-COOH: 0.2, 0.1	THC: 0.1–100 THC-COOH: 0.1–100	[138]
LC-MS	Plastic tube	500 µL of expectorated OF	SPE	None	THC: 2, 5	THC: 5–2000	[89]
2D-GC-MS (NICI for THC-COOH)	Quantisal	1 mL of Quantisal OF	SPE	BSTFA TFAA (for THC-COOH)	THC: 0.5 11-OH-THC: 0.4, 0.5 THC-COOH: 6, 7.5 CBD: 0.5 CBN: 1	THC: 0.5–50 11-OH-THC: 0.5–50 THC-COOH: 7.5–500 CBD: 0.5–50 CBN: 1–50	[139]
LC-MS/MS	Intercept	400 µL of Intercept OF	SPE	None	THC: 0.2 THC-COOH: 0.2	THC: 0.25–8 THC-COOH: 0.25–8	[140]
LC-MS/MS	Quantisal	1 mL of Quantisal OF	SPE	Triphenylphosphine, 2-picolylamine and 2,2′-dypyridyl disulfide	THC: 0.6, 1 THC-COOH: 6, 10	THC: 1–100 THC-COOH: 10–1000	[141]
LC-MS/MS (quadrupole/orbital)	Plastic tube	400 µL of OF in preservation buffer	Liquid-liquid extraction and SPE	None	THC: 2 (1 point calibration) THC-COOH: 7.5	THC-COOH: 7.5–300	[102]
LC-MS/MS	Plastic tube	200 µL of expectorated OF	Liquid-liquid extraction	None	THC: 1	THC: 1–500	[142]
LC-MS/MS	Plastic tube	250 µL of expectorated OF	Dilute and shoot	Dansyl chloride	THC: 0.005, 0.025 THC-COOH: 2.5	THC: 0.2–20	[143]
LC-MS/MS	Plastic tube	250 µL of expectorated OF	SPE	None	THC: 0.1 11-OH-THC: 0.1 THC-COOH: 0.1 CBD: 0.1 CBN: 0.1	THC: 0.1–50 11-OH-THC: 0.1–50 THC-COOH: 0.1–50 CBD: 0.1–50 CBN: 0.1–50	[144]
LC-HRMS	Oral-Eze, Quantisal	250 µL of Oral-Eze and 500 µL of Quantisal OF	SPE	None	THC: 0.5 THC-COOH: 9, 12 CBD: 0.5 CBN: 0.5	THC: 0.5–50 THC-COOH: 12–1020 CBD: 0.5–50 CBN: 0.5–50	[145]
LC-MS/MS	Quantisal	1 mL of Quantisal OF	SPE	None	THC-COOH: 9, 12	THC-COOH: 12–1020	[146]
LC-MS/MS	Plastic tube	225 µL of expectorated OF	MEPS	None	THC: 0.08, 0.25 11-OH-THC: 0.12, 0.4 THC-COOH: 8, 20 CBD: 0.1, 0.3 CBN: 0.12, 0.3	THC: 0.25–250 11-OH-THC: 0.4–250 THC-COOH: 20–1000 CBD: 0.3–250 CBN: 0.3–250	[147]
LC-MS/MS	StatSure, Quantisal	100 µL of StatSure 200 µL of Quantisal or Certus OF	Liquid-liquid extraction	None	THC: 5	THC: 5–320	[113]
LC-MS/MS	Quantisal	1.5 µL of combined Quantisal sample/methanol extract	SPE	None	THC: 0.3, 0.5 11-OH-THC: 0.2, 0.5 THC-COOH: 50, 80 CBD: 0.3, 0.5 CBN: 0.3, 0.5 THCAA	THC: 0.5–75 11-OH-THC: 0.5–75 THC-COOH: 50–500 CBD: 0.5–75 CBN: 0.5–75	[148]
GC-MS/MS	Quantisal, Oral-Eze	1 mL of Quantisal or 750 µL of Oral-Eze OF	SPE	HFIP and TFAA	THC-COOH: 7.5, 10	THC-COOH: 10–1000	[149]
LC-MS/MS	Quantisal	1 mL of Quantisal OF	SPE	None	THC: 0.1, 0.2 11-OH-THC: 0.1, 0.2 THC-COOH: 15 CBD: 0.1, 0.2 THCV CBG	THC: 0.2–100 11-OH-THC: 0.2–50 THC-COOH: 15–3750 CBD: 0.2–50	[123]

Abbreviations: 11-OH-THC: 11-hydroxy-tetrahydrocannabinol; 2D-GC-MS: 2D gas chromatography-mass spectrometry; BSTFA: N,O-bis(trimethylsilyl)trifluoroacetamide; CBD: cannabidiol; CBG: cannabigerol; CBN: cannabinol; GC-MS/MS: gas chromatography-tandem mass spectrometry; GC-MS: gas chromatography-mass spectrometry; HFIP: 1,1,1,3,3,3-hexafluoro-2-propanol; LC-HRMS: liquid chromatography-high resolution mass spectrophotometry; LC-MS/MS: liquid chromatography-tandem mass spectrometry; LC-MS: liquid chromatography–mass spectrometry; LC-QTOF-MS: liquid chromatography-quadrupole time-of-flight mass spectrometry; MEPS: microextraction by packed sorbent; PFAA: pentafluoro propionic anhydride; SPE: solid-phase extraction; TFAA: trifluoroacetic anhydride; THC: Δ^9^-tetrahydrocannabinol; THCAA: Δ^9^-tetrahydrocannabinol acid; THC-COOH: 11-nor-9-carboxy-tetrahydrocannabinol; THCV: tetrahydrocannabivarin.

**Table 7:** Detection of cannabinoids in oral fluid specimens in on-site point-of-care biosensors [36,103].

Manufacturer (City, Country)	Device	Year	Interpretation of Result	Device Cutoff (THC ng/mL)	Laboratory Cutoff (ng/mL)	Oral Fluid Confirmation	Sensitivity (%)	Specificity (%)	Accuracy (%)	Refs.
Cozart (Abingdon, UK)	RapiScan	2007	Instrumental	600	-	HPLC/GC-MS	-	100	100	[102]
	Cozart DDSV	2009	Visual	-	0.5	GC-MS	41.2	100	60	[150]
	Cozart DDS 806	2011	Instrumental	31	1	UPLC-MSMS GC-MS	22	100	71	[151]
		2012	Instrumental	31	10	UPLC-MSMS	28.2	100	78.7	[51]
	Cozart DDS	2012	Instrumental	31	1	UPLC-MSMS GC-MS	37.8	100	94.3	[152]
Mavand (Eschweiler, Germany)	RapidSTAT	2010	Visual/Instrumental	15	1.6	GC-MS	85	87	86.7	[153]
		2011	Visual	15	1	GC-MS UPLC-MSMS	68 56	89 90	86 78	[154] [151]
		2012	Visual	15	1 2 * 10	GC-MS GC-MS UPLC-MSMS	72 71 43.3	97 55 88.3	93 66 78.2	[152] [155] [51]
Biosensor (München, Germany)	BIOSENSE Dynamic	2011	Instrumental	Unknown	1	UPLC-MSMS GC-MS	50	Not reported	51	[151]
Sun Biomedical (Blackwood, NJ, USA)	OraLine	2006	Visual	4	1	HPLC/GC-MS	69	92	74	[156]
	OraLine IV	2007	Visual	100	1	HPLC/GC-MS	100	36	54.3	[102]
Varian (Palo Alto, CA, USA)	OraLab	2007	Visual	100	1	HPLC/GC-MS	40	100	76	[102]
	OraLab	2007	Visual	1	2	LC-MS	93.3	98.6	98.1	[157]
	OraLab 6	2011	Visual	50	1	UPLC-MSMS GC-MS	16	99	61	[151]
Innovacon (San Diego, CA, USA)	OrAlert	2011	Visual	100	1	UPLC-MSMS GC-MS	11	100	78	[151]
	OrAlert	2012	Visual	100	10	UPLC-MSMS	23.1	100	90.9	[51]
Branan (Irvine, CA, USA)	Oratect	2007	Visual	100	1	HPLC/GC-MS	0	100	77.8	[102]
	Oratect III	2011	Visual	40	1	UPLC-MSMS GC-MS	32	100	41	[151]
American Bio Medica (Kinderhook, NY, USA)	OralStat	2007	Visual	25	1	HPLC/GC-MS	70	100	91.4	[102]
LifePoint (Ontario, CA, USA)	Impact	2007	Instrumental	15	1	HPLC/GC-MS	100	33.3	71.4	[102]
Ulti-Med (Ahrensburg, Germany)	SalivaScreen	2007	Visual	>100	1	HPLC/GC-MS	-	100	100	[102]
OraSure Technologies (Bethlehem, PA, USA)	Uplink	2007	Instrumental	25	1	HPLC/GC-MS	100	92	95.6	[102]
Securetec (Neubiberg, Germany)	DrugWipe	2007	Visual	30	1	HPLC/GC-MS	80	100	82.9	[102]
	DrugWipe 5	2008	Visual	30	2	GC-MS	52	91	85	[158]
	DrugWipe	2011	Visual	30	1	GC-MS	43	96	88	[154]
	DrugWipe	2012	Visual	30	1	GC-MS	47	99	93	[152]
	DrugWipe5/5^+^	2011	Visual	30	1	GC-MS	43	87	82	[159]
	DrugWipe 5A	2016	Visual	30	0.6 ng/pad	HS-SPME/GC-MS	29	88	53	[160]
	DrugWipe 5	2010	Visual	30	2 *	GC-MS	71	50	63	[155]
	DrugWipe 5^+^	2013	Visual	30	Unknown	GC-MS	88	94	88	[161]
Dräger (Lübeck, Germany	DrugTest 5000	2006	Instrumental	20	0.5	LC–MS–MS	53	94	55.5	[162]
	DrugTest 5000	2010	Instrumental	20	2 *	GC-MS	82.5	60.5	79	[155]
	DrugTest 5000	2011	Instrumental	5	1	UPLC-MSMS GC-MS	59	96	82	[151]
	DrugTest 5000	2012	Instrumental	5	Unknown	GC–MS	91	43	85.5	[161]
	DrugTest 5000	2012	Instrumental	5	0.5	2D-GC-MS GC-MS	87.7	81.2	85.5	[102]
	DrugTest 5000	2012	Instrumental	5	1	GC-MS	92	97	97	[152]
	DrugTest 5000	2012	Instrumental	5	10	UPLC-MSMS	81	96	92	[51]
Alere (North Chicago, IL, USA)	DDS 2	2017	Instrumental	25	1	LC–MS/MS	90	100	97.5	[163]

Abbreviations: OF—oral fluid; THC–Δ^9^—tetrahydrocannabinol; 2D-GC—2-dimensional GC; GC—gas chromatography; HPLC—high-performance LC; LC—liquid chromatography; MS—mass spectrometry; UPLC—ultraperformance LC. * blood was used as a reference.

## Data Availability

Not applicable.
